# How to publish a new fungal species, or name, version 3.0

**DOI:** 10.1186/s43008-021-00063-1

**Published:** 2021-05-03

**Authors:** M. Catherine Aime, Andrew N. Miller, Takayuki Aoki, Konstanze Bensch, Lei Cai, Pedro W. Crous, David L. Hawksworth, Kevin D. Hyde, Paul M. Kirk, Robert Lücking, Tom W. May, Elaine Malosso, Scott A. Redhead, Amy Y. Rossman, Marc Stadler, Marco Thines, Andrey M. Yurkov, Ning Zhang, Conrad L. Schoch

**Affiliations:** 1 Department of Botany and Plant Pathology, Purdue University, West Lafayette, IN 47907 USA; 2 Illinois Natural History Survey, University of Illinois Urbana-Champaign, Champaign, IL 61820 USA; 3 Genetic Resources Center, National Agriculture and Food Research Organization, 2-1-2 Kannondai, Tsukuba, Ibaraki 305-8602 Japan; 4 Westerdijk Fungal Biodiversity Institute, Uppsalalaan 8, 3584CT Utrecht, the Netherlands; 5 State Key Laboratory of Mycology, Institute of Microbiology, Chinese Academy of Sciences, NO.1 Beichen West Road, Chaoyang District, Beijing, 100101 China; 6 Comparative Plant and Fungal Biology, Royal Botanic Gardens, Kew, Surrey, TW9 3DS UK; 7 Department of Life Sciences, The Natural History Museum, Cromwell Road, London, SW7 5BD UK; 8 Jilin Agricultural University, Changchun, 130118 Jilin Province China; 9 Center of Excellence in Fungal Research, Mae Fah Luang University, Chiang Rai, 57100 Thailand; 10 Biodiversity Informatics & Spatial Analysis, Royal Botanic Garden Kew, Richmond, London, TW9 3AE UK; 11 Botanischer Garten und Botanisches Museum, Freie Universität Berlin, Königin-Luise-Str. 6-8, 14195 Berlin, Germany; 12 Royal Botanic Gardens Victoria, Birdwood Avenue, Melbourne, Victoria 3004 Australia; 13 Departamento de Micologia, Centro de Biociências, Universidade Federal de Pernambuco, Recife, PE 50740-600 Brazil; 14 Ottawa Research and Development Centre, Science and Technology Branch, Agriculture and Agri-Food Canada, Ottawa, Ontario K1A 0C6 Canada; 15 Botany and Plant Pathology Department, Oregon State University, Corvallis, OR 97333 USA; 16 Department Microbial Drugs, Helmholtz Centre for Infection Research, Inhoffenstrasse 7, 38124 Braunschweig, Germany; 17 Department of Biological Sciences, Institute of Ecology, Evolution and Diversity, Goethe University, Max-von-Laue-Str. 13, 60438 Frankfurt am Main, Germany; 18 Senckenberg Biodiversity and Climate Research Centre, Senckenberganlage 25, 60325 Frankfurt am Main, Germany; 19 Leibniz Institute DSMZ-German Collection of Microorganisms and Cell Cultures, Braunschweig, Germany; 20 Department of Plant Biology, Rutgers University, New Brunswick, NJ 08901 USA; 21 National Center for Biotechnology Information, National Library of Medicine, National Institutes of Health, 45 Center Drive, Bethesda, MD 20892 USA

**Keywords:** Data repositories, Dual nomenclature, FAIR principles, Legitimate publication, New combinations, Taxonomic best practices, Typification, Valid publication

## Abstract

It is now a decade since *The International Commission on the Taxonomy of Fungi* (ICTF) produced an overview of requirements and best practices for describing a new fungal species. In the meantime the *International Code of Nomenclature for algae, fungi, and plants* (ICNafp) has changed from its former name (the *International Code of Botanical Nomenclature*) and introduced new formal requirements for valid publication of species scientific names, including the separation of provisions specific to *Fungi* and organisms treated as fungi in a new Chapter F. Equally transformative have been changes in the data collection, data dissemination, and analytical tools available to mycologists. This paper provides an updated and expanded discussion of current publication requirements along with best practices for the description of new fungal species and publication of new names and for improving accessibility of their associated metadata that have developed over the last 10 years. Additionally, we provide: (1) model papers for different fungal groups and circumstances; (2) a checklist to simplify meeting (*i*) the requirements of the ICNafp to ensure the effective, valid and legitimate publication of names of new taxa, and (*ii*) minimally accepted standards for description; and, (3) templates for preparing standardized species descriptions.

## INTRODUCTION

Scientific names are an essential link that serve to communicate biological information across many spheres of use. The Linnaean system, which is central to all scientific names, dates to the eighteenth century. Its central tenet of a hierarchical classification predates modern concepts of evolution and phylogenetic systematics yet has endured as an adaptable and intuitive system to name and classify organismal diversity. The use of binomial names for species, drawing on Latin and latinized ancient Greek, is a legacy that has been incorporated into requirements for the description of new species, known as the Codes or Rules of nomenclature, which vary between groups of organisms and are developed by internationally mandated bodies.

The relevant *Code* for *Fungi* as well as non-fungal groups traditionally treated as fungi, such as oomycetes and slime moulds, is the *International Code of Nomenclature for algae, fungi, and plants* (ICNafp, or simply, the *Code*). The ICNapf is revised at Nomenclature Section meetings of each, now six-yearly*, International Botanical Congress* (IBC)—except for fungal groups, where changes that apply only to them form a separate Chapter F that is decided upon at each, four-yearly, *International Mycological Congress* (IMC). The current ICNafp and Chapter F are, respectively, those of Turland et al. (2018; https://www.iapt-taxon.org/nomen/main.php) and May et al. (2019).

For a new name to be validly published and thus available for use there are formal (or ‘legal’) requirements that are imposed by the *Code*, termed Articles. For example, the designation of a type accompanied by a diagnosis and/or a description of the species is mandated by the *Code*. A type designation serves to link the name for a given species to a physical entity (a specimen, metabolically inactive culture, or in special cases, an illustration), which is selected to represent that name. A type specimen then serves as the standard for future comparative analyses.

In addition to following the nomenclatural rules set out by the *Code*, and the Recommendations made in them, biologists are expected to thoroughly document newly proposed species in a manner designed to facilitate identification and data accessibility by users. These actions are distinguished from the formal requirements of nomenclature and reside in the separate domain of taxonomy. Thus, while there are no formal rules for taxonomic description, there are nonetheless community standards of scientific rigor enforced by journal editors and reviewers, which should be adhered to when publishing names of new taxa.

*The International Commission on the Taxonomy of Fungi* (ICTF) first published a paper designed to give guidance and provide best practices for authors proposing new species in 1987 (Sigler and Hawksworth 1987), which was updated in 2010 (Seifert and Rossman 2010). The purpose of the present paper is to provide mycologists with freshly updated guidelines for publication of new species incorporating: (1) changes to the *Code*; and (2) updated best practices that have emerged since 2010. We divide our focus into two areas: (*i*) nomenclature, providing a clear set of guidelines for valid publication as set out in the *Code*; and (*ii*) taxonomy, proposing a set of minimal standards or best practices that we recommend for documentation in a publication and for data submission to public repositories. Finally, we provide a checklist for ensuring publications meet formal nomenclatural requirements and best practices, examples of model papers, and a template for standardizing new taxon descriptions. In taxonomy, no single set of standards can apply to all fungal groups and all circumstances, but it is incumbent on authors to adhere to standards set for their taxonomic group and to justify deviations from these. With the guidelines and suggestions as set forth in this paper, we hope to aid in the publication of valid, acceptable, and useful names of new fungal taxa. We also intend to promote easily accessible data associated with these actions to improve usage of taxonomic names and accelerate scientific discovery.

## NOMENCLATURE

### Formal requirements

The goal of nomenclature is to stabilize the usage of names via an internationally agreed set of rules that are known as a *Code.* The most recently published *Code* is the one which must be followed as each new *Code* or Chapter F renders the previous one obsolete. New provisions in the *Code* are also retroactive, unless specifically limited by date. Provisions specific to fungi have been separated into Chapter F of the ICNafp (https://www.iapt-taxon.org/nomen/pages/main/art_f5.html). After each IMC, a revised Chapter F is published online in *IMA Fungus* that supersedes the previous version; the most recent Chapter F includes changes approved at IMC11 in San Juan, Puerto Rico, in July 2018 (May et al. 2019). Details specific to fungi covered in Chapter F include sanctioning (Art. F.3), protection via lists of names (Art. F.2.1), and registration of names and typification acts (Art. F.5). The *Code* is a highly technical document, but examples are provided to demonstrate the application of the rules it contains. A general account of the *Code* is now published as *The Code Decoded* (Turland 2019, https://ab.pensoft.net/article/38075/), which provides an explanatory version of the formal *Code* and includes examples for fungal names.

The most important and relevant changes made to the *Code* since the publication of Seifert and Rossman (2010) are summarised here:

1. ***The elimination of dual nomenclature****.* It is no longer acceptable to apply two or more scientific names for a single fungus, including the use of different names for different morphs of the same species. One can, however, use informal terms such as hypocrea-like sexual morph in describing a new species of *Trichoderma* (syn. *Hypocrea*) or tubercularia-like asexual morph for a new species of *Nectria* (syn. *Tubercularia*)*,* rather than the previously used generic names *Hypocrea* and *Tubercularia.*
2. ***The elimination of a Latin requirement.*** Latin is no longer required for descriptions and/or diagnoses, and these can now be either in English or Latin but not in any other language (Art. 39.2).
3. ***Registration of nomenclatural novelties****.* Nomenclatural novelties (e.g. new taxa, replacement names, or new combinations) must be accompanied by citation of an identifier number obtained from a repository that has been appointed by the *Nomenclature Committee for Fungi* (NCF) and ratified by an IMC. Every new name published must have a separate identifier. Thus, when a new genus name is published simultaneously with one or more new species, each new name (genus and species) must have separate identifiers (Art. F.5.1). At the time of writing, the recognised repositories are Fungal Names (http://www.fungalinfo.net/), Index Fungorum (http://www.indexfungorum.org) and MycoBank (https://www.mycobank.org). It is only necessary to obtain a single identifier from one of these repositories for each nomenclatural novelty. The standard method of citing the registration identifier is to place the number after the name of the relevant repository, in full and/or as an acronym (FN, IF or MB), separated by a space (see example manuscript template, below).
4. ***Registration of new typification acts.*** As of 1 Jan 2019, any new type designation, such as a lectotypification, neotypification, or epitypification, for previously described taxa at or below the rank of species must be registered and the identifier cited with the typification act (Art. F.5.4).
5. ***Types of cultured fungi.*** As of 1 January 2019, where a type is derived from a living culture, the method of preservation must be clearly indicated as being “metabolically inactive” (Art. 40.8) to be acceptable as the type.
6. ***Cross Code homonyms***. Homonyms are names of families, genera or species spelled exactly the same. If a name is a later homonym it is illegitimate (Art. 53). From 1 January 2019, names of fungi must not only avoid being homonyms of names of other organisms covered by the *Code*, but also avoid repeating prokaryotic or protozoan names governed by other codes (Art. F.6.1).

#### Effective, valid, and legitimate publication

In order to be available for use, names for new taxa must be ‘effectively’, and ‘validly’ published and be ‘legitimate’. These three words have precise meanings in nomenclatural terminology and they should not be used in other ways in taxonomic manuscripts. These terms are defined in the Glossary to the *Code* as well as in *The Code Decoded* (Turland 2019).

(1) ***Effective publication*** **(Arts. 29–31)**. Effective publication concerns the kinds of publications in which names of new taxa can be published. As of 1 January 2012, effective publication can include publishing in online-only formats but must adhere to certain requirements including that the online document is available in Portable Document Format (PDF) and has an International Standard Serial Number (ISSN) or an International Standard Book Number (ISBN), as explained in Turland (2019). When publishing online, we discourage placing nomenclatural novelties and new typifications in supplementary material as these may not be readily discoverable. In addition, online supplementary material is not effectively published unless it is in PDF format and explicitly linked to a publication with an ISBN or ISSN (Art. 29.1).

(2) ***Valid publication*** **(Arts. 32–45)**. For a name of a new taxon to be validly published, it must meet the requirement for effective publication, and meet the additional provisions laid out for valid publication in the current *Code*. These include provisions for correct designation of holotypes or basionyms, and registration of names. If a published name is not valid, such as due to omission of an identifier, such a name may be validated at a later time and then will date from the time of validation. Invalidity of names is the most common issue with new names, and the process to subsequently validate names can be complicated. Therefore, close attention to all requirements for valid publication is advisable. Note that if a name was not validly published but has been issued an identifier, either in the protologue or by a repository, its subsequent validation requires a separate, new identifier.

(3) ***Legitimate publication*** **(Art. 6)**. In addition to meeting the requirements for both effective and valid publication, a new name must be legitimate in accordance with the rules in Art. 6.5. Illegitimate names include homonyms (i.e., a name spelled exactly the same as a previously published name), or a new name that includes the type of a previously published available name or epithet that should have been adopted.

#### Designation of a type

Designation of a type, to serve as a physical reference for a name, is one of the most important aspects of valid publication (Art. 40). The purpose of a type is to fix the application of the name (Art. 7.1), that is, to serve as a reference of a species for contemporary and future researchers from which direct comparisons to other living and preserved specimens can be made. New species must include the designation of a *holotype* specimen and this must be deposited in a single specified institution. If more than one fungarium/herbarium/culture collection is designated for the holotype, the name is invalid. In addition to a holotype, other kinds of type specimens may be appropriately designated under certain circumstances. *Isotypes* are genuine duplicates of the holotype that may be deposited in the same repository that holds the holotype or, preferably, in additional repositories. *Epitypes* (Art. 9.9) may be designated and attached to an existing type when the original type material lacks sufficient characters to be unambiguously identified. When designating an epitype, a reference must be made to the type that it supports. The words “designated here” must be included for *epitype, lectotype* and *neotype* designations. Best practices for type selection are discussed separately below.

## TAXONOMY

### Best practices

Biological taxonomy is the science of classifying objects or organisms. It embraces identifying, describing, and classifying organisms by comparing them to known taxa. The goal of taxonomic best practices is to: (1) provide all necessary data needed for both contemporary and future workers to identify taxa and use associated data in downstream analyses; and (2) reduce the likelihood of publication of taxonomically superfluous names. Unlike the *International Code of Nomenclature of Prokaryotes*, the ICNafp does not require that descriptions should conform to any minimal standards for *Code*-compliant publication (ICNP Rec.30; Parker et al. 2019). Nonetheless, taxonomists are increasingly playing the role of intermediaries when it comes to linking biodiversity information, published literature and online databases. Therefore, they are responsible for communicating their data so that it can be verified, shared broadly, and used by other scientists (Durkin et al. 2020; Lücking et al. 2020). In this section we make recommendations for the minimal and, preferably, maximal accepted standards, that will allow repeatability and re-examination of any published study and its analyses.

The first, and perhaps most important step to take when preparing to describe a new taxon, is to ensure that a name does not already exist for it. It is important to bear in mind that one should not infer a new species by absence of sequence matches in public repositories. Only a small fraction of described fungal species are represented in public repositories, and, depending on the group, an estimated 30% of these sequences can be misidentified (Hofstetter et al. 2019). Detailed discussion of this topic is beyond the scope of the current paper, although adherence to the guidelines suggested herein and in Dayarathne et al. (2016) will lessen the probability of creating superfluous synonyms.

#### Describing new species

The key element of a description is the demonstration that a specimen or culture represents a species that is distinct from previously described species. Authors of new species should bear in mind that the goal of communicating new species is to make identities clear and unambiguous for current users and future generations. To facilitate this goal, best practices should include: multiple collections, where possible, to account for and describe phenotypic and, where applicable, genotypic, variation within a species; use of multiple data types, where feasible, for clear delineation of species; and provision of tools such as DNA barcode data to facilitate rapid identification. While it will not be possible to adhere to these guidelines in all situations (Hawksworth 2020), author diligence in meeting as many of these recommendations as possible will ensure the publication of high-quality and discoverable data.

1. ***Number of collections.*** When describing a new species, it is always preferable to include multiple collections of specimens or cultured isolates. In special circumstances, such as for rare taxa, taxa from highly specialized niches or remote locales, fossilized fungi, or non-culturable microfungi, multiple collections may not be possible. In these instances, it is recommended that authors provide multiple lines of evidence that could include, for example, multi-locus DNA analyses, gross morphology, and physiological or biochemical data for designation of new species. Authors should also demonstrate diligence in attempting to obtain additional material by providing, for example, information about the sampling methods used. Publication of cryptic species based on a single collection, without strong supporting evidence, is discouraged.
2. ***Minimally accepted evidence for a new species.*** New species hypotheses, even those with multiple collections, should be supported by evidence drawn from more than one kind of data. These could include, but are not limited to, any of the following: macro- and micromorphology, multi-locus DNA analyses, metabolic or proteomic data (e.g., secondary metabolite profiles, assimilation data, mass spectrometry), physiology, ecology, biogeography, mating studies, or taxon-specific approaches. Phylogenetic data alone may be insufficient evidence for designation of new species, and recommendations for their use are outlined further below. It is important to not only incorporate different data sources but also to document different stages of the life cycle of a fungus, particularly in lineages with complex life cycles or multiple morphs. For instance, evidence of due diligence in searching for sexual reproductive structures should be provided for asexual species.
  It is good practice, when dealing with fresh material, to attempt to isolate the fungus in pure culture. This is valuable not only so that the culture can be deposited in a culture collection and, thus, be available for future experimental work, but culturing single ascospores or basidiospores, for example, potentially allows the discovery of a morph not previously seen in the original collection. In some taxa cultures are mandatory, for example where certain growth characteristics under standard conditions are used for species delineations (e.g. *Penicillium*).
3. ***Species concepts.*** Although it is recommended to employ a combination of several experimental methods, referred to as a polyphasic approach or integrative taxonomy, for delimiting species, authors should provide a statement of the guiding species concept used to delimit newly proposed species (Lücking et al. 2020). Because the best species concepts to apply can vary, authors of new species should be familiar with the concept(s) that have been tested and applied to their group. One recommended strategy is to consult taxonomic experts for a given group or consider their collaboration in the description of new species.
4. ***Minimally accepted ecological and geographic information*.** Habitat refers to the ecosystem from which the material was collected (e.g. coniferous forest, lake, coffee plantation). Substrate is what the material was found growing on (e.g. wood, oak leaf, soil). Only use the term “host” to describe symbionts (pathogens to mutualists) isolated directly on or from within another organism (e.g. *Quercus alba*). When the ecology is unknown this should be indicated (Durkin et al. 2020). Geographic information should ideally include sampling coordinates, altitude, climate (temperatures or a climate classification), and description of the biotope (including dominating organisms); for soil-borne species it is important to specify soil type according to FAO WRB (IUSS Working Group 2015). Geographic coordinates should be given as decimal degrees to facilitate their use in further analyses.
5. ***Publication of DNA barcode sequences.*** It is recommended that, where possible, DNA barcode sequence(s) be provided in a public repository, minimally, for the holotype specimen or ex-type strain. Public repository accession numbers should be included with the type designation as part of the deposition statement. We recommend generation and publication of the fungal barcode locus (ITS, Schoch et al. 2012) as well as any additional taxon-specific secondary barcode loci.
6. ***Compliance with the Convention on Biological Diversity and the Nagoya Protocol.*** The 1992 UN Convention on Biological Diversity (CBD) and one of its supplementary agreements, the 2010 Nagoya Protocol (NP), aim to promote biodiversity conservation, ensure sustainable use of biodiversity, and enable fair and equitable benefit sharing from use of biodiversity. Of particular relevance in this respect is the recognition of the sovereign right of individual countries to genetic resources within their borders and the statement that no such materials should be removed without agreement between the collector of samples and the competent authority in the country of origin of the genetic resources. Because type collections should be removed from their environment and deposited in a single institution (Art. 40.7), the following regulations should always be considered: (1) legal sampling; (2) legal deposition; and (3) utilisation of (research on) the collected material. Although it may sound trivial, it is important to remember that sampling permits should be obtained before collecting samples. Following national CBD/NP regulations, many public collections (fungaria/herbaria for preserved (dead) material and biological resource centres for live cultures) check to ensure that all new deposits adhere to the legislation and request a copy of relevant sampling and/or research permits. It is recommended that authors also include a statement about compliance with CBD/NP in the methods or acknowledgements. While the CBD and NP represent international regulations, most countries also have separate legal requirement for collecting and research, even if the country did not ratify the CBD (for example, the USA) or if the national NP regulation does not apply to a particular territory (Greenland). We encourage taxonomists to engage in collaboration with colleagues in the target country.

**New combinations, names at new rank, and replacement names:** It is strongly recommended that publication of new combinations include evidence of the taxonomic decisions and the types of the basionyms (or replaced names) should be examined before making any changes and cited. A statement should be provided as to why the species should not be classified in the former genus and is more appropriately classified in the new genus. When combining species into different genera, include notes on how to differentiate the newly recombined species from other similar species in that genus.

**Typification:** A type serves as the standard for future comparative analyses and should aim to be selected as a typical representative of the species. Holotypes should be selected, where possible, from a collection with adequate material for generating DNA barcode(s) and for designating additional isotypes. When original type material of previously published names is poor or ambiguous, new combinations may benefit from the designation of an epitype. Guidelines for determining whether epitypification is warranted have been published elsewhere (Ariyawansa et al. 2014). Wherever possible, epitypes should be designated from the same geographic area and habitat, substrate, or host as the original type. Only a holotype, lectotype or neotype (Art. 9.9) can be epitypified, and when designating an epitype, a reference must be made to the holo−/lecto−/neotype that the epitype supports. In cases where the original holotype specimen is lost, and no original material (or original illustration) is available for lectotypification, a neotype should be designated that should be in a condition comparable to an epitype, i.e. should allow the accurate interpretation of the species.

**Diagnosis and description:** The *Code* no longer requires the publication of both a diagnosis and a description, although at least one or the other must be included. Nevertheless, ICNapf recommends the use of both, as they serve different purposes (Rec. 38B.1).

1. ***Diagnosis****.* A diagnosis is a short statement that delineates the new taxon from similar described taxa. A diagnosis should aim to provide a succinct statement about the key diagnostic character(s) for the accurate identification of the species.
2. ***Description****.* The goal of a description, as opposed to a diagnosis, is to provide a complete and detailed account of the characters of the new taxon. Data to include in a description vary between taxonomic groups, but the overall goal is to provide enough taxon-specific detail for a user to unambiguously confirm an identification. In general, descriptive data follow broadly established standards for different taxonomic groups (see suggested model papers). The kinds of data will include a complete morphological/phenotypic description derived from examination of multiple specimens within multiple collections or isolates where possible; known geographic range and hosts or substrate preferences; and other taxon-specific data, such as publication of assimilation panels in yeasts or metabolic profiles.
3. ***Images and/or illustrations*.** Inclusion of a photo plate or illustrations to indicate clear morphological/phenotypic features is usually required, and strongly recommended, to support the descriptions. Authors should provide high quality images of new species that could include images of the general habitat, including close-ups, and of diagnostic anatomical features, such as sections, culture characteristics, or spores. Non-diagnostic anatomical features that are uniform within a larger group of species need not be illustrated. In order to decide which images to include, authors should bear in mind that subsequent researchers should be able to readily assess characters based on the description and images, without having to restudy the type material. When registering a new taxon, adding images to the registration is also recommended, especially if the protologue is published in a journal without open access.
4. ***Notes.*** Notes are used to expand on the diagnosis, for instance by providing a broader taxonomic discussion of the new taxa relative to a larger number of similar and/or related species and rationale for why a taxon is considered new. They are also the place to include any nomenclatural remarks. Underlying taxonomic work(s) from which comparative data are taken should be cited.

**Species authors citations:** Species authors citations, i.e., the author names for a new taxon, also follow naming conventions. The names of authors of fungal taxa follow standardized form, typically abbreviations, a complete list of which can be found on *Index Fungorum* (http://www.indexfungorum.org/names/AuthorsOfFungalNames.asp) and the *International Plant Names Index* (IPNI) (https://www.ipni.org/). First time authors of new names should check *Index Fungorum* or IPNI to ensure that the abbreviation chosen for their name is not already in use. Subsequent new taxa should use the same abbreviation for any given author. If an author changes their name, e.g. by marriage, they should keep their original name for new taxa. Equally important is determining the authors to be included on a taxon name. These may or may not be the same as the authors of the paper in which the new taxa are described. Taxon authors should be chosen with the same consideration as the authors of the paper, i.e., should consist of those individuals who were directly involved in collecting and/or determining the identity and status of the new taxon. Superfluous or gratuitous authors leading to long lists of authors after a taxon name should be avoided. Also bear in mind that community practice is that honorifics, i.e., new taxa named after a specific living person, should not be published in papers in which the honored person is a co-author.

**Choosing a publication:** Online-only publication in journals and formats is now permitted for effective publication (but see above). However, we recommend that authors publish only in peer-reviewed publications, and that new names include detailed descriptions, illustrations, and notes, even when not required by the publishing venue.

**Use of molecular data:** The application of molecular data in phylogenetic analyses to delimit taxa has become a widespread, beneficial practice and may be one of the best ways to accurately circumscribe cryptic taxa or species in complexes. In most cases, use of phylogenetic evidence should be one of multiple types of data employed to support recognition of a taxonomic novelty. Publication of names of new species based solely on single-locus phylogenetic analyses should only be done in exceptional cases, such as where prior studies have demonstrated their utility for discriminating species in the same lineage. Recommendations for minimal information to ensure repeatability and accuracy of inferences drawn from phylogenetic analyses are as follows:

1. **Number of sequenced representatives**. Wherever possible, multiple sequenced representatives for a new taxon from different collections or isolates should be included in the analyses, including, minimally, the type specimen. In special circumstances where this may not be possible, it is recommended that sequencing of the respective DNA loci be repeated to ensure accuracy of data.
2. **Number of sequenced loci.** Where possible, taxonomic inferences should be based on multi-locus analyses. Single locus analyses in support of data drawn from numerous other lines of evidence may be appropriate, provided locus selection is in keeping with those previously demonstrated to provide adequate resolution for the taxa under consideration. While the ITS barcode region is the preferred locus for *identification*, it is seldom the preferred locus for species *delimitation* (Lücking et al. 2020). We do not recommend the general use of ITS alone for the delimitation and description of new species, unless already demonstrated to have the appropriate resolving power for the group to which it is being applied. Use of appropriate loci for phylogenetic reconstruction and species delimitation varies by taxonomic group and authors should invest effort in researching and applying the most appropriate loci for their group. The use of genomic data in phylogenomic analyses may meet the requirement for multiple loci but should still fulfill the recommendations for appropriate taxon sampling outlined in (3) below.
3. **Sampling strategy for phylogenetic analyses.** We recommend that an explicit statement about taxon selection/sampling strategy be included in the methods section of a paper that applies phylogenetic data to taxon delimitation. Even with multiple sequences from different collections and multiple loci or genomes, analyses without appropriate context can result in inaccurate inference. Inclusion of representative sequences from the type species of a genus or genera treated are strongly recommended, as are sequences of closely related or similar taxa. Use of tests of genealogical concordance in delimiting cryptic species should be applied where feasible, including the publication of the single locus phylogenies (for instance in supplementary material) in addition to the concatenated tree. Where sequences used in analyses are derived from public repositories, the underlying papers that generated those data should be cited.

### Best practices for data accessibility

**Physical data**: Although it is not mandated in the *Code*, it is strongly encouraged that types are lodged in publicly accessible, recognized biorepositories. Type material should not be deposited in private/personal fungaria/herbaria/culture collections. Where possible, additional type material such as isotypes, paratypes, or ex-type cultures should be deposited in multiple institutions. Always ensure that the biorepository is correctly registered in *Index Herbariorum* (Thiers 2020) or the *World Directory of Culture Collections* (http://www.wfcc.info/). Authors depositing material collected from foreign countries should be able to demonstrate that the material was collected in compliance with the NP and meets any host-country stipulations about deposition of material (see above; Smith et al. 2017; Yurkov et al. 2019). Recommendations for best practices for deposition of types include:

1. **Selecting a repository***.* When possible, ensure that the repository is one in which access to materials via loan or other means is easily obtained and freely available. Wherever possible, we recommend deposition of one or more isotypes or paratypes in other repositories, ideally held within different continents or countries, especially if the holotype institution is not readily accessible.
2. **Culture collections**. Living actively propagated material, such as actively metabolizing cultures, cannot serve as a holotype, but must be preserved in a metabolically inactive state, such as by cryopreservation or lyophilization in culture collections, or as dried cultures kept in fungaria/herbaria. For culturable fungi the *Code* recommends preservation of duplicate living cultures prepared from the holotype strain in at least two publicly accessible culture collections (Rec. 8B.1), ideally residing in more than one country or continent. Certified repositories and bioresource centres (BRC) that follow rigorous quality standards are recommended. Where additional material derived from the holotype culture are stored metabolically inactive, and specified as such, they are isotypes rather than ex-type cultures. Living strains derived from the holotype isolate should be included in the type information designated as ex-holotype culture/s, with their repository and accession number/s. We do not recommend use of a superscript capital letter T as a method of indicating a type in the holotype statement, as the use is ambiguous. Use of an equal (=) sign to link strains derived from the type culture is also discouraged. See example type citation for a type that is a culture in the model manuscript section, below.

**Digital data:** Submission of data including both specimen metadata and molecular sequence data to public online repositories has become an integral part of modern species descriptions and the amount of biodiversity represented in the public sequence databases is increasing rapidly (Schoch et al. 2020). Large online aggregators of voucher specimen (e.g. *Atlas of Living Australia*, GBIF, iDigBio, MyCoPortal, *Species*Link) and culture (e.g. CBS, UAMH, ATCC) metadata provide essential resources for highlighting new species discoveries beyond the original publication and for linking the different components (specimen, culture, and sequence) of the extended specimen (Lendemer et al. 2020). A central principle in modern data usage is the need to make these data Findable, Accessible, Interoperable and Reusable, codified as the FAIR data principles (Wilkinson et al. 2016). This principle is embraced by major data warehouses such as GBIF, iDigBio and the partners of the *International Nucleotide Sequence Database Collaboration* (INSDC; Karsch-Mizrachi et al. 2018), which includes the *DNA Data Bank of Japan* (DDBJ), the *European Nucleotide Archive* (ENA) and GenBank at the *National Center for Biotechnology Information* (NCBI).

Several of these recommendations have already been made specifically to address sequence records from the commonly used ITS barcode marker (Nilsson et al. 2012) and we expand on this in a more general sense here. Specimen voucher and strain metadata should always be populated for INSDC records. This means listing the place of deposition (see above for guidelines in selecting these). If the institution code is correctly provided, this enables metadata to be harvested directly from the institution database. This is only true as long as its holdings are digitised and available online. If the data are not online elsewhere, they should be provided in full. Do not combine voucher information from multiple isotypes or co-identical strains in a single sequence record; rather refer to the individual specimen voucher from which the sequence data were derived. More detailed suggestions are made below:

1. ***Specimen metadata submissions***. Depositing specimens with richly populated, Darwin Core (DwC) formatted metadata in fungaria/herbaria/culture collections that share their records online not only helps to legitimize the new taxa but allows users to easily find, access and verify material without having to search through numerous publications. At a minimum, specimen metadata should include, where known: 1) country, state/province, municipality, and locality; 2) habitat, substrate and host (if applicable); 3) decimal latitude and longitude; 4) elevation in meters; 5) date of collection and, if a culture, date of isolation; 6) collector(s) and collection number; and 7) fungarium/herbarium acronym and barcode or accession number (see Table 1). Information routinely required by culture collections is similar, and all these fields except for 4 and 7 are mandatory for deposition (Boundy-Mills et al. 2016). Once specimen record data are available online, they can be linked to and from nomenclatural repositories (*Fungal Names, Index Fungorum*, *MycoBank*), genetic sequence databases (e.g. *GenBank*), citizen scientist websites (i.e. *Mushroom Observer*, *iNaturalist*), and ecological portals (e.g. *FunGuild*). Linking specimen data with ancillary, machine-readable data in online public formats extends the usefulness of the new species and enhances the discovery of new fungal taxa.

(2) ***Sequence submissions***.
  - Any sequence marker should be clearly labelled according to its commonly used locus or gene name. Sequences intended to be submitted as DNA barcodes should only include the generally used region for that marker in order to allow BLAST searches to work optimally. For example, submissions for the ITS region (Schoch et al. 2012) should be submitted with only short SSU and LSU flanking sequences (Moncada et al. 2020).
  - Trim low-quality ends of sequences. Sequence quality is influenced by these variable ends and it often affects quality checks, increasing the likelihood of annotation errors for introns etc., which can mean that some marker sequences are tagged as unverified and thus excluded from certain database searches.
  - Ensure that the entire sequence is submitted, and not just the aligned region that may have variable regions excluded.
  - Follow the instructions stipulated by the INSDC member database you are using. Please be aware that not all fields are shared by all three members.
  - Voucher information associated with public sequence records should be structured following DwC data standards to enable indexing of vouchers and their biorepositories (Schoch et al. 2020). An initial search in the NCBI BioCollections database (Sharma et al. 2018) should yield information on the unique code to use. This database incorporates information from other, more extensive resources*. Index Herbariorum* is the reference for institutional acronyms of fungaria/herbaria (Thiers 2020; Index Herbariorum, http://sweetgum.nybg.org/science/ih/). For culture collections the *World Directory of Culture Collections* (CCINFO) (http://www.wfcc.info/) and *World Data Center for Microorganisms* (http://www.wdcm.org/) provide standardised abbreviations. Most fungaria/herbaria/culture collections do not separate their holdings into internal collections, and hence the common usage is only two components, for example “NY:1234” or “CBS:1234”. In some cases, a mycological collection will have a separate entry and it is recommended to verify this in NCBI BioCollections. For example, the fungarium of the Royal Botanic Garden Kew uses K(M), which would be followed by the accession number as “K(M):123456”.
  - Specimen records can be linked to/from GenBank via the LinkOut feature provided by NCBI, for example the ITS-LSU sequence record of *Cortinarius wiebeae* (https://www.ncbi.nlm.nih.gov/nuccore/KF732479) links directly to the holotype specimen from the provided LinkOut links (https://mycoportal.org/portal/collections/individual/index.php?occid=343584) in MyCoPortal, which links their ITS sequence back to the GenBank record.
  - For brief recommendations for the most common qualifiers at NCBI see Table 1. Do not add duplicate voucher information such as isotypes or co-identical strains; any voucher information should be reserved for the specimen/culture from which the sequences were generated. Any of this additional info can be added as a note.

**Table 1:** Most commonly used modifiers recommended for public biodiversity sequence records

NCBI qualifier	Note (INSDC controlled vocabulary link listed where applicable)	Example
collected_by	Name of person who collected the sample, please use initials and surname.	/collected_by = “A.H. Smith”
collection_date	Day, month and year when the sequenced specimen was collected.	/collection_date = “23-Aug-1948”
country	Country where the sample was collected. Additional region or locality information must be after the country name and separated by a ‘:’. See http://www.insdc.org/documents/country-qualifier-vocabulary	/country = “USA: Washington, Pierce County, Mt. Rainier National Park”
culture_collection	Format for cultures in culture collections: ‘institution-code:culture-id’. culture-id and institution-code are mandatory. When possible use code documented in NCBI BioCollections or WFCC.	/culture_collection = “CBS:1752”
host	Use full verified binomial, if possible. Incomplete names such as genus sp. is acceptable.	/host = “*Quercus longinux*”
isolate	Use this for lab numbers/ field numbers of the specific specimen/culture from which this sequence was obtained.	/isolate = “JT13209”
isolation_source	Reserved for physical or environmental source and substrate information.	/isolation_source = “dead wood”
lat_lon	Latitude and longitude, in decimal degrees, of where the sample was collected.	/lat_lon = “28.721667 N 17.785278 W”
note	Add any additional unstructured information such as isolation method, isotype info not addressed in the other fields.	/note=”“DNA isolation: REPLI-g Single Cell Kit (Qiagen)”
specimen_voucher	Format for dried specimens: ‘institution-code: internal-code:specimen-id’. specimen-id is mandatory. When possible use code documented in NCBI BioCollections or Index Herbariorum or indicate personal herbaria by adding in front:‘personal:’ See http://www.insdc.org/controlled-vocabulary-specimenvoucher-qualifier	/specimen_voucher = “MICH:14410” or /specimen_voucher = “MICH:AH Smith 30,553” or /specimen_voucher = “personal: AH Smith 30,553”
strain	Use this for strain numbers of pure cultures, i.e. those not deposited in culture collections.	/strain = “ABC 1234”
tissue_type	Ignore this field unless it refers to source tissue information e.g. blood, skin etc..	
type_material	This field is not user submitted - it is automatically updated only after the publication or nomenclature database entry is verified by NCBI Taxonomy curators. Please provide the full publication as a pdf to gb-admin@ncbi.nlm.nih.gov. (Do NOT use the Type modifier for this information). See http://www.insdc.org/controlled-vocabulary-typematerial-qualifer	/type_material = “holotype of *Tuber anniae*”

### Models for preparing new species manuscripts and descriptions

**Fig. 1:**
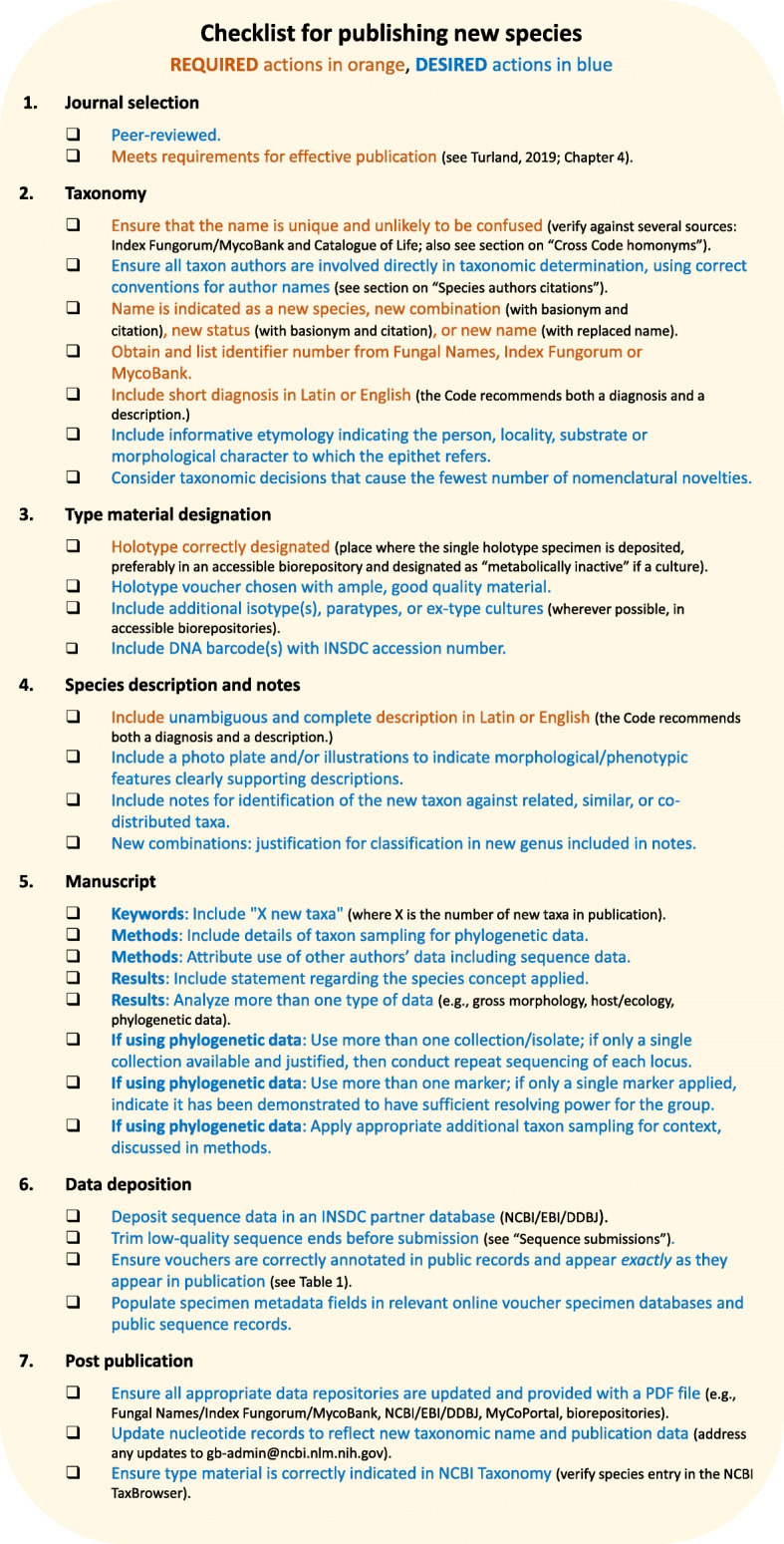
Checklist for publishing new species

**Checklist for publishing new species:** Fig. 1.

**Use of keywords:** Any paper publishing one or more taxonomic novelties should include the keywords “X new taxa” to aid online indexing databases, where “X” stands for the number of new taxa introduced (Schoch et al. 2017).

**Published examples for new species following best practices:** Several model examples for describing different kinds of new species and names are provided below.

1. *New species of sporulating fungi.* (Niveiro et al. 2020. 10.3897/mycokeys.66.4871; Réblová et al. 2020. 10.3897/mycokeys.74.57824)
2. *New species of cultured fungi including yeasts.* (Walsh et al. 2021, 10.1080/00275514.2020.1803649; Haelewaters et al. 2020, 10.3114/fuse.2020.05.12; Santos et al. 2020, 10.1002/yea.3453; Aime et al. 2018, 10.1080/00275514.2018.1446650; Bezerra et al. 2017, 10.1007/s11557-016-1254-0; Toome et al. 2013, 10.3852/12-251; Gimánez-Jurado et al. 2003, 10.1099/ijs.0.02470-0)
3. *Species that are sterile (only known from vegetative structures) (*i.e. *most endophytic fungi).* (Noumeur et al. 2020, 10.1007/s11557-020-01581-9; Wibberg et al. 2021, 10.1007/s13225-020-00447-5; Koch et al. 2018, 10.1007/s11557-018-1411-8; Knapp et al. 2015, 10.3767/003158515X687669)
4. *New species of parasitic*/*pathogenic fungi.* (Liu et al. 2020, 10.1080/00275514.2020.1781496; Luo et al. 2017, 10.1080/00275514.2017.1400306; Aoki et al. 2013, 10.3852/12-262; Edwards et al. 2016, 10.3852/15-333)
5. *New species of lichenized or lichenicolous fungi.* (Lendemer 2021, 10.1639/0007-2745-124.1.090; Spribille et al. 2020, 10.1017/S0024282920000079; Diederich et al. 2019, 10.2478/pfs-2019-0021; Lücking et al. 2017, 10.1007/s13225-016-0374-9)
6. *New species of fossil fungi.* (Pound et al. 2019, 10.1080/01916122.2018.1473300)
7. *New cryptic fungal species.* (Kruse et al. 2018, 10.5598/imafungus.2018.09.01.05)
8. *New species based on taxon-specific characters.* (Kuhnert et al. 2017, 10.1007/s13225-016-0377-6)
9. *New species based on molecular diagnoses*. (Linde et al. 2017, 10.5598/imafungus.2017.08.01.03)
10. *New combinations.* (Hernández-Restrepo et al. 2020, 10.3114/fuse.2020.06.01; Luo and Zhang 2013, 10.3852/12-359)
11. * Revival and application of “old” names.* (Wittstein et al. 2020, 10.1016/j.simyco.2020.01.001; Minnis et al. 2012, https://doi.org/10.3852/11-350)
12. *Epitypification.* (Mighell et al. 2021, 10.1080/00275514.2020.1816386; Lendemer 2020, https://onlinelibrary.wiley.com/doi/full/10.1002/tax.12289)

**Example submissions in GenBank:**

https://www.ncbi.nlm.nih.gov/nuccore/MH040548.1

https://www.ncbi.nlm.nih.gov/nuccore/NR_111529.1

Please note that the second example is a NCBI RefSeq entry that was selected by an NCBI curator from a GenBank record and in this case received additional appended metadata, although the sequence data remain unchanged (Robbertse et al. 2017).

**Example template for a new species description:**

***Mynew species*** Auth. and Auth2, sp. nov. **Fig(s)** xxxx [cite those included].

[One of] Fungal Names/Index Fungorum/MycoBank: FN/IF/MB XXXXXX.

**Etymology**: Explain the origin and meaning of the new species name (Art. 23). Bear in mind the ease of pronouncing and/or spelling names when selecting. See Stearn (1992) for Latin versions of descriptive terms and geographic regions or place names.

**Diagnosis** [English or Latin]: Similar to *Xxxxx xxxx* but differs in some key/diagnostic character(s).

[Ex. 1—type is a non-culture specimen] **Type:** COUNTRY: State/Province, Municipality/County/District, Locality; Geographic coordinates (in decimal degrees), altitude (in metres). Biome/habitat, substrate/host. Date (00 Month 0000) of collection/isolation. *Collector(s) collector number*. (**Holotype** FUNGARIUM/HERBARIUM XXXX; **isotype** FUNGARIUM/HERBARIUM XXXX; [where applicable] ex-holotype culture CULTURE_COLLECTION XXXX). GenBank/ENA/DDBJ: XXXXXXXX (ITS); XXXXXXXX (Other_locus).

[Ex. 2—type is a culture] **Type:** COUNTRY: State/Province, Municipality/County/District, Locality; Geographic coordinates (in decimal degrees), altitude (in metres). Biome/habitat, substrate/host. Date (00 Month 0000) of collection/isolation*. Collector(s) collector number*. (**Holotype** FUNGARIUM/HERBARIUM/CULTURE_COLLECTION XXXX, stored in a metabolically inactive state; **isotype** FUNGARIUM/HERBARIUM/CULTURE_COLLECTION XXXX, stored in a metabolically inactive state; ex-holotype culture CULTURE_COLLECTION XXXX, CULTURE_COLLECTION XXXX). GenBank/ENA/DDBJ: XXXXXXXX (ITS); XXXXXXXX (Other_locus).

**Description** [English or Latin]: This section will contain a complete description including:

Sexual morph (where present).

Asexual morph (where present).

Taxon-specific descriptors—e.g., assimilation tests, biochemical analyses, gross culture morphology, optimal growth temperatures. Refer to photographs and/or illustrations that supplement descriptions.

**Ecology/Substrate/Host**: This section will provide a concise statement of the known ecology, including biome, habitat, and substrate/hosts.

**Distribution**: This section will provide a concise statement of distribution of the new taxon as is currently known. This may include data retrieved from public repositories, such as ITS blasts from GenBank, UNITE or GlobalFungi, that show an expanded range beyond the actual material examined.

**Material examined**: A list of all other additional collections/isolates of *Mynew species*. Format follows the same as in holotype designation. Where appropriate, it may also include a second paragraph listing non con-specific collections examined that were also compared to *Mynew species* to delimit the new species and should include other type specimens or generic types consulted for comparison.

**Notes**: These will include any additional notes about the new taxon. These typically consist of: (1) A brief summary statement on the rationale of the new taxon, including considerations on the underlying data and their limitations. (2) A comparative taxonomic discussion expanding on the diagnosis, where *Mynew species* is compared to all similar and related species such as those similar in morphology, and similar species that share the same niches or hosts, are co-distributed, and/or are phylogenetically closely related. Includes citations of underlying taxonomic references from which comparative data were obtained. (3) Where necessary, a paragraph on nomenclatural aspects, in particular whether types of available names have been studied to make sure the new species does not have an earlier available name. (4) Other notes regarding the new taxon, such as unique characteristics or potential uses.

**Example template for a new combination description:**

***Mynew combination*** (Auth.) Auth2, comb. nov.

[One of] Fungal Names/Index Fungorum/MycoBank: FN/IF/MB XXXXXX.

**Basionym**: original name and a full and direct reference given to its author and place of valid publication, with page (or plate reference) and date. Refer to ICNafp Art. 30.3 for proper citation under different circumstances.

[Where necessary] **Typification:** Lecto/Neo/Epitype designated here: COUNTRY: State/Province, Municipality/County/District, Locality; Geographic coordinates (in decimal degrees), altitude (in metres). Biome/habitat, substrate/host. Date (00 Month 0000) of collection/isolation. *Collector(s) collector number*. (**Lecto/Neo/Epitype** FUNGARIUM/HERBARIUM XXXX; Typification_identifier [One of] Fungal Names: FN XXXXXX -OR- Index Fungorum: IF XXXXXX -OR- MycoBank: MBT XXXXXXXX). GenBank/ENA/DDBJ: XXXXXXXX (ITS); XXXXXXXX (Other_Locus). In the case of an epitypification, the supported holo/lecto/neotype also needs to be indicated.

**Description**: This section will contain a re-description if there are new data to report. Or may contain a reference to other published descriptions and illustrations.

**Substrate/Host**: As above, if there are new data to report.

**Distribution**: As above, if there are new data to report.

**Material examined**: As above. Include type specimens examined that have informed the new combination.

**Notes**: These will outline why *Mynew combination* belongs in the *Mynew* genus and not in the prior one as well as a brief comparison to similar or related species in the new genus. Where a lecto/neo/epitypification is necessary the reasons for the typification act should be given.

## CONCLUSIONS

It should always be borne in mind that introducing a new scientific name is a responsibility, as it will remain in databases to be taken into account by all future researchers—even if it proves to be not validly published or a synonym of an already known species. The present contribution does not consider various taxonomic pitfalls that can be encountered when describing novel fungal species, some of which have recently been pointed out elsewhere (Hawksworth 2020). Neither does it address issues surrounding the application of previously published names and their typification, which can sometimes be complex, especially for names published in the 18th and 19th centuries in the absence of internationally agreed rules (Dayarathne et al. 2016). The ICTF is therefore planning a complementary guide to best practices in dealing with older names, and model papers (no. 11, above) are supplied for guidance. Finally, the ICTF is planning a guide to additional issues related to collection and storage of fungal vouchers and cultures that will expand on the recommendations in the current paper.

In this paper, we have provided a list of formal requirements for effective, valid and legitimate publication of new taxa. In addition, recommendations are made for author responsibilities, such as providing adequate descriptive data and figures, attribution of underlying data used in comparisons, and accurate annotation of data in public databases. Ideally, we would recommend that journals adopt at least the minimal required practices suggested here. Previously, the ICTF has published recommended best practices on reporting keywords for novel taxa (Schoch et al. 2017) and on use of italicization (Thines et al. 2020) and these have been adopted by many journals and are included in the author guidelines. We provide several tools such as a checklist and templates for ensuring that published data meet both the requirements of the *Code* and community best practices. We also encourage the publication of molecular data, especially DNA barcode data, for new species. As mentioned earlier, the naming of lineages known only from sequence data is not currently addressed by the *Code* but practical solutions remain under discussion within the fungal community (Lücking et al. 2021). While no single set of recommendations will be applicable across all fungi and under all circumstances, it is incumbent on authors to provide as much high-quality data as possible for future users. By following these guidelines, including the important but often overlooked step of updating repositories upon publication, taxonomists will allow for independent verification of taxonomic hypotheses and ensure that data management facilitates downstream use and access to accurate biodiversity information and metadata.

## Data Availability

Data sharing is not applicable to this article as no datasets were generated or analysed specifically for this purpose.
